# Effects of Hybrid POSS Nanoparticles on the Properties of Thermoplastic Elastomer-Toughened Polyamide 6

**DOI:** 10.1021/acsomega.3c06896

**Published:** 2023-11-29

**Authors:** Rumeysa Yıldırım, Muhammad Saeed Ullah, Hürol Koçoğlu, Merve Ün, Nazlı Yazıcı Çakır, Gülşah Demir, Duygu Çetin, Gizem Urtekin, Güralp Özkoç, Olcay Mert, Mehmet Kodal

**Affiliations:** † Polymer Science and Technology Graduate Programme, Kocaeli University, 41001 Kocaeli, Türkiye; ‡ Chemical Engineering Department, Kocaeli University, 41001 Kocaeli, Türkiye; § Mechanical Engineering Department, Bolu Abant Izzet Baysal University, 14030 Bolu, Türkiye; ∥ Nanotechnology Research and Application Center SUNUM, Sabanci University, 34956 İstanbul, Türkiye; ⊥ Chemistry Department, İstinye University, 34010 İstanbul, Türkiye; # Chemistry Department, Kocaeli University, 41001 Kocaeli, Türkiye

## Abstract

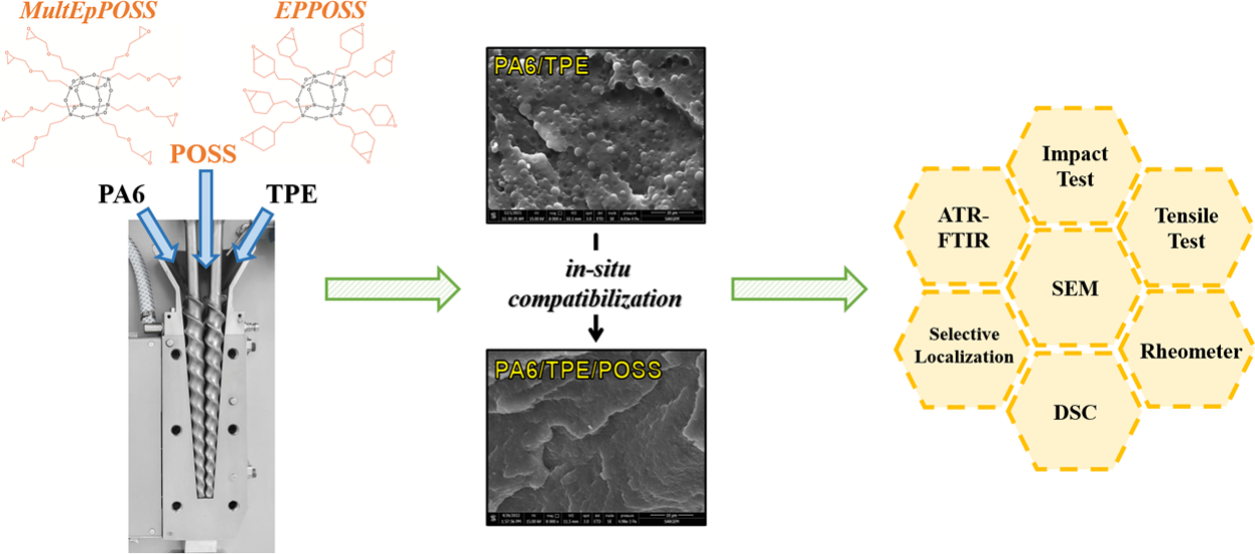

In this study, polyamide 6 (PA6)/thermoplastic elastomer (TPE) blends were prepared to decrease the notch sensitivity of PA6 for automotive applications, and the morphological, rheological, mechanical, and thermal properties of PA6/TPE blends, which are partially miscible or immiscible depending on the TPE ratio, were significantly improved in the existence of polyhedral oligomeric silsesquioxane (POSS) nanoparticles with multiple reactive epoxy groups as compatibilizers. An unstable phase morphology was obtained with the addition of TPE into PA6 without POSS nanoparticles, whereas interfacial interactions between phases in the presence of POSS were enhanced as a result of a significant decrease in the average particle size from 1.39 to 0.41 μm. The complex viscosity value of the 70PA6/30TPE blend, which was 20 kPa/s^–1^ at 0.1 rad/s angular frequency, reached 380 kPa/s^–1^ with the addition of POSS due to the formation of long chains by the generation of graft and/or block copolymers, which resulted in a 65% increase in Young’s modulus value. Most notably, the Izod impact strength of pure PA6, which was 10 kJ/m^2^, increased by 290% with the incorporation of POSS. It was confirmed by FTIR analysis that the reactive multiple epoxy groups of MultEpPOSS and EPPOSS nanoparticles react with the proper groups of PA6 and/or TPE, and also, a partial hydrogen bonding interaction occurs between PA6-TPE from the shifting of N–H and carbonyl peaks. In conclusion, it can be suggested that POSS nanoparticles can serve as highly effective compatibilizers for PA6/TPE blends and have potential commercial applications, especially in the automotive sector.

## 1 Introduction

Polyamide 6 (PA6) is an engineering thermoplastic with many important commercial applications due to its superior properties such as high chemical, abrasion, corrosion, and fatigue resistance, high melting temperature (*T*_m_), and toughness. However, notch sensitivity, low notched impact toughness, high moisture absorption, poor impact resistance below the glass transition temperature (*T*_g_), and poor dimensional stability limit the application of PA6.^1−10^ These disadvantages of PA6 can be overcome by blending with other polymers, especially elastomers.^11−13^

Recently, thermoplastic elastomers (TPEs) have been used to toughen polyamides. TPEs are polymeric materials that can be called bridging materials between rubber and plastics. Additionally, these materials can give final properties like rubber and can also be processed like thermoplastics.^14,15^ These materials consist of a flexible segment, often called the amorphous part, which has a low *T*_g_, and a hard crystalline segment, which has a higher *T*_g_ than the amorphous part.^16,17^ Due to the physical interactions between the hard and soft segments, these materials are thermally unstable, causing them to flow like a thermoplastic at high temperatures. Their unique properties, such as price/performance ratio, lightweight, high mechanical properties, and easy processing like thermoplastics, have made them one of the most important materials nowadays.^18−20^ Polyester thermoplastic elastomers are one of the most important types of TPEs. Polyester-based TPEs are a segmental copolyether formed by melt transesterification of dimethyl terephthalate, a polyalkylene ether diol, and a low-molecular-weight diol. The crystallizable long tetramethylene terephthalate hard segments act as a cross-linking agent. Due to the network bonding of these hard segments to the soft phase polyalkylene ether glycol teraphthalate, the whole system behaves like a cross-linked elastomer. Although polyester-based TPEs show high tensile strength at high temperatures, they have low permanent deformation, chemical resistance, and thermal stability at elevated temperatures.^21^

As with almost all polymer blends, PA6/TPE blends have been reported in the literature to be incompatible and form multiphase systems due to transcrystallization.^22,23^ This is due to the fact that polyamide crystallizes before the crystallizable phase of TPE, causing phase separation.^24^ The final properties of thermodynamically immiscible polymer blends depend on the morphology of the system and the interfacial adhesion between the phases.^12^ Due to thermodynamic instability, immiscible polymer blends exhibit unsatisfactory physical properties, as they have poor dispersion and poor interfacial interaction. Therefore, reactive compatibilizers are often used to improve the compatibility of thermodynamically immiscible polymer blends.^25,26^ In this method, either block or graft copolymers are added to the blend during blending, or such copolymers are obtained in situ during blending. The compatibilizers act as emulsifiers in the interphase, reducing the interfacial tension.

In recent years, polyhedral oligomeric silsesquioxane (POSS) nanoparticles have come to the forefront due to their flexible physical and chemical properties and their economical use on an industrial scale. POSSs are structurally cage-shaped molecules. They can be perceived as polyhedral skeletons formed by silicon and oxygen with the closed formula (RSiO_1.5_)*_n_*. Here, “*n*” is greater than 4 and often 8. The R group in the structure can consist of many different functional groups. In this way, the chemical and physicochemical properties of POSSs, such as solubility and reactivity, in different polymer matrices can be tuned. POSS molecules have a diameter of 1.5 nm and a molecular weight of approximately 1000 Da. Therefore, POSS molecules are almost equal to the molecular size of many polymer chains.^27^ POSSs facilitate the dispersion of the polymer at the molecular level within the matrix when appropriately chosen to be compatible and to interact (physical or chemical) with the matrix, yielding a nanocomposite. Although such a situation is ideal, it is generally seen in the literature that POSSs exhibit a dispersion in the 100–500 nm range.^28^ Notable improvements in mechanical and thermal properties, as well as thermal resistance, can be seen in polymer/POSS composite systems when dispersion takes place at the nanoscale and interactions with the polymer matrix are developed.

There are many studies in the literature on blending polyamide with elastomers to obtain new materials with high-impact resistance.^2,29,30^ Yu et al. investigated the styrene–ethylene–butadiene–styrene block copolymer (SEBS), ethylene-1-octene copolymer (POE), ethylene-vinyl acetate rubber (EVA), and their maleated derivatives (SEBS-*g*-MAH, POE-*g*-MAH, and EVA-*g*-MAH) as impact modifying agents for PA1010. The findings showed that adding more maleated elastomer led to a decrease in the size of the dispersed phase’s particles, whereas adding an elastomer to a polyamide increased its impact strength values.^31^ Jeziórska et al. investigated the effect of polyethylene functionalized with ricinol-2-oxazoline methyl maleate (PE-*g*-MRO) as a compatibilizer in the compatibilization of PA6/thermoplastic polyester elastomer blends. They observed that interactions between the functional groups of the thermoplastic polyester elastomer PA6 and PE-*g*-MRO resulted in the creation of a compatible heterogeneous structure by reactive extrusion.^32^ In another study, melt blends of PA6 and the polyester elastomer were prepared in the presence of 1,4-phenylene bis(2-oxazoline) (PBO) and diglycidyl ether bisphenol (DGEBA) coupling agents. The end-chain groups of the polyester elastomer and the terminal amide groups of PA6 were predicted to react with the oxazoline groups of PBO and the epoxy groups of DGEBA, resulting in the reactive extrusion of copolymers. The findings showed that the coupling agents used in the study increased the compatibility between PA6 and the polyester elastomer, resulting in a more stable phase morphology.^13^ Majumdar et al. used maleic anhydride-grafted SEBS-*g*-MAH for the compatibilization of PA6/SEBS blends. It was stated that the particle size of SEBS decreased in the PA6 matrix due to reactive compatibilization in the presence of SEBS-*g*-MAH.^33^ Another research investigated the effects of SEBS modified with various maleic anhydride concentrations on the ternary blends of PA6/SEBS/maleated SEBS. The findings demonstrated that employing maleated SEBS improved the impact strength of pure PA6 by 30 times.^34^

In this work, the compatibilizing efficiency of epoxy-based POSS nanoparticles in PA6/TPE blends with different ratios of immiscible and partially miscible components was examined for the first time in the literature. The motivation behind selecting epoxy-based POSS molecules as compatibilizers stems from the potential for the reaction between the amide (–NHCO–), amine (–NH_2_), and/or carboxylic acid (–COOH) groups of PA6 and the hydroxyl (–OH) and/or carboxylic acid (–COOH) groups of TPE and the epoxy groups attached to the cage structure of POSS. For this purpose, multiple epoxy group-containing aliphatic (MultEpPOSS) and cycloaliphatic (EPPOSS) POSS types were selected. As a result, the compatibilizing effectiveness of POSS nanoparticles was carefully examined in terms of their chemical composition and characteristics. Depending on the POSS loading ratio, the morphological, rheological, chemical, mechanical, and thermal characteristics of PA6/TPE blends were investigated. Additionally, to establish the dispersion regions of POSS nanoparticles inside polymer blends, selective localization studies were performed.

## 2 Materials and Methods

### 2.1 Materials

Polyamide 6 (PA6; trade name: Tecomid NB60 NL) was obtained from Eurotec, Türkiye. A polyester-based thermoplastic elastomer (TPE; trade name; Arnitel UM552 TPC-ES) was supplied from DSM, The Netherlands. Glycidyl POSS (MultEpPOSS) and epoxycyclohexyl ethyl POSS (EPPOSS) were procured from Hybrid Plastics Company. POSSs are in the viscous liquid state at room temperature. The chemical structures of materials used in this study are shown in Table 1.

**Table 1:**
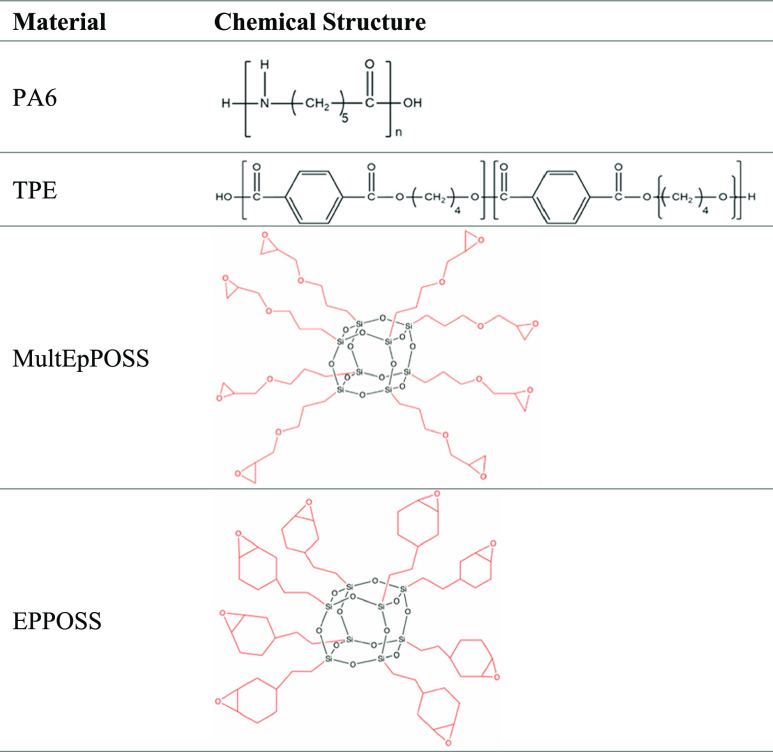
Chemical Structures of Materials

### 2.2 Sample Preparation

Prior to compounding, PA6 and TPE were dried in a vacuum oven at 80 °C for 12 h to remove potential moisture. The samples were then prepared via melt blending using a laboratory-scale twin-screw microcompounder (15 mL microcompounder, MC 15 HT, Xplore Instruments, The Netherlands). The operating parameters of the microcompounding process were 100 rpm screw speed and 230 °C barrel temperature. The residence time of the materials in the melt blending process was 2 min. To avoid thermooxidative degradation, the barrel was continuously purged with nitrogen gas during melt blending. After the residence time, the melt was molded using a laboratory-scale microinjection molding device (12 mL injection molder, IM 12, Xplore Instruments, The Netherlands) to produce test samples according to ISO 527–2/5A and ISO 180 standards. The injection pressure was kept at 10 bar, while the melt and mold temperatures were set at 230 and 25 °C, respectively. The components and ratios of PA6/TPE blends and sample coding are given in Table 2.

**Table 2:** Components and Ratios of PA6/TPE Blends

sample	PA6 ratio (wt %)	TPE ratio (wt %)	POSS ratio (wt %)
PA6	100	0	0
TPE	0	100	0
70PA6/30TPE	70	30	0
70PA6/30TPE/0.5MultEpPOSS	70	30	0.5
70PA6/30TPE/1MultEpPOSS	70	30	1
70PA6/30TPE/0.5EPPOSS	70	30	0.5
70PA6/30TPE/1EPPOSS	70	30	1
50PA6/50TPE	50	50	0
50PA6/50TPE/0.5MultEpPOSS	50	50	0.5
50PA6/50TPE/1MultEpPOSS	50	50	1
50PA6/50TPE/0.5EPPOSS	50	50	0.5
50PA6/50TPE/1EPPOSS	50	50	1

### 2.3 Characterization

#### 2.3.1 Fourier Transform Infrared Spectroscopy (FTIR)

Attenuated total reflectance-Fourier transform infrared spectrometry (ATR-FTIR) analyses were performed by using a PerkinElmer spectrum 100 FTIR instrument. The samples were analyzed in the wavenumber range of 4000–650 cm^–1^.

#### 2.3.2 Selective Localization Measurements

Selective localization analyses were performed to determine the exact positions of POSS nanoparticles within PA6/TPE blends. Contact angle analyses were used to investigate the localization of the nanoparticles within the polymer matrix. The total surface energy of a nonmetallic material (γ_i_^TOT^) can be divided into two parts: Liftshitz–van der Waals component (γ_i_^LW^) and acid–base component (γ_i_^AB^). Details of the calculation of these components can be found in our previous study.^35^ The surface energy components of the probe liquids (diiodomethane, ethylene glycol, and deionized water) and probe solids (polypropylene (PP), PA6, and polystyrene (PS)) used in this study are shown in Table 3.

**Table 3:** Surface Energy Components of the Probe Liquids and Solids

	probe liquids^36^			probe solids^a^		
surface energy (mJ/m^2^)	diiodomethane	ethylene glycol	deionized water	PP	PA6	PS
γ_i_^TOT^	50.8	48.0	72.8	30.1	44.4	40.7
γ_i_^LW^	50.8	29.0	21.8	30.1	36.3	34.6
γ_i_^AB^		19.0	51.0		8.5	6.1
γ_i_^–^		47.0	25.5		12.1	2.2
γ_i_^+^		1.9	25.5		1.5	0.1

a Obtained experimentally.

Contact angle measurements were carried out using the KSV Attention Theta device. The contact angle values obtained from the samples were used to identify the polymer–filler and polymer–polymer interfacial tensions, and then, the wettability parameters were calculated. At least five repetitions were performed for each sample to ensure the repeatability of the measurements. Three probe solids were used to determine the surface free energy components of MultEpPOSS and EPPOSS nanoparticles in the liquid form. The surface energy components of the POSSs were calculated based on the surface energy values of the probe solids and the contact angle measurement results obtained.

#### 2.3.3 Scanning Electron Microscopy (SEM)

The phase morphologies of PA6/TPE blends were investigated by using a QUANTA FEG 450 scanning electron microscope (SEM). SEM images were obtained from the cryogenically fractured surfaces of the impact specimens. Before analysis, the samples were coated with a thin layer of gold to eliminate arching. The average particle size of the dispersed phase (*d*_AVG_) was determined using image analysis software (ImageJ).

#### 2.3.4 Rheological Analyses

Rheological properties were determined by using an Anton Paar MCR 102 rheometer with parallel plate geometry. Frequency sweep measurements were performed under a nitrogen atmosphere at a constant temperature of 230 °C. The angular frequency range varied between 0.1 and 600 rad/s while keeping a shear strain of 0.1%.

#### 2.3.5 Tensile Test

The tensile properties of the specimens were evaluated using an Instron (Model 3345) universal testing machine according to ISO 527–2/5A. The crosshead speed was set as 50 mm/min.

#### 2.3.6 Impact Test

Notched Izod impact strengths of the specimens were measured by a Ceast Resil Impactor according to the ISO 180 standard.

#### 2.3.7 Differential Scanning Calorimetry (DSC) Analyses

Differential scanning calorimetry (DSC) analyses were performed under a nitrogen purge by using the Mettler Toledo DSC1 Star System. The samples were subjected to a heating rate of 10 °C/min at a 25–250 °C heating range. The samples were kept at 250 °C for 5 min to eliminate any thermal history. The samples were then cooled from 250 to 25 °C at a cooling rate of 10 °C/min and then reheated to 250 °C at a heating rate of 10 °C/min.

## 3 Results and Discussion

### 3.1 Fourier Transform Infrared Spectroscopy (FTIR)

**Figure 1:**
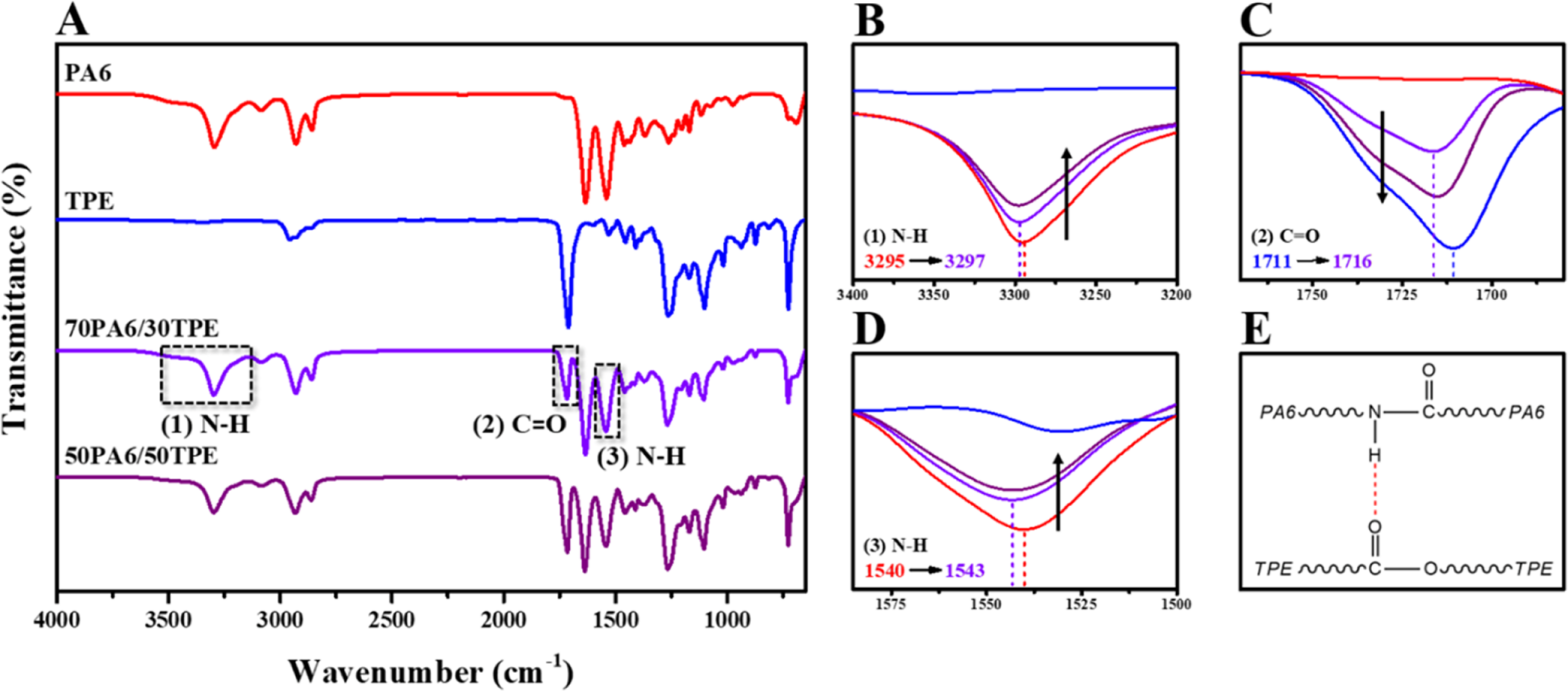
ATR-FTIR spectra of neat polymers and PA6/TPE blends (A). The enlargement of the (1) N–H region (B), (2) C=O region (C), and (3) N–H region (D). Formation of a hydrogen bond between PA6 and TPE (E).

The characteristic peaks of pure PA6 and TPE along with the changes in corresponding peaks in PA6/TPE blends having 70/30 and 50/50 compositions were determined by spectroscopic analysis by ATR-FTIR (Figure 1A–D). As a result of the decrease in PA6 content with the addition of TPE to PA6, the intensities of the N–H peaks ((1) and (3)) of PA6 decreased (Figure 1B,D), whereas the intensity of C=O peak (2) of TPE increased (Figure 1C). Moreover, the N–H stretching vibration at 3295 cm^–1^^37^ and N–H bending vibration at 1540 cm^–1^^38^ of pure PA6 slightly shifted to 3297 and 1543 cm^–1^, respectively, upon addition of 30% TPE to PA6 (70PA6/30TPE blend; Figure 1B,D), and the ester carbonyl (C=O) stretching vibration at 1711 cm^–1^ of PBT^39^ in pure TPE significantly shifted to 1716 cm^–1^ in the 70PA6/30TPE blend (Figure 1C), proving the hydrogen bond formation between the N–H protons of PA6 and the carbonyl (C=O) groups of TPE (Figure 1E).

**Figure 2:**
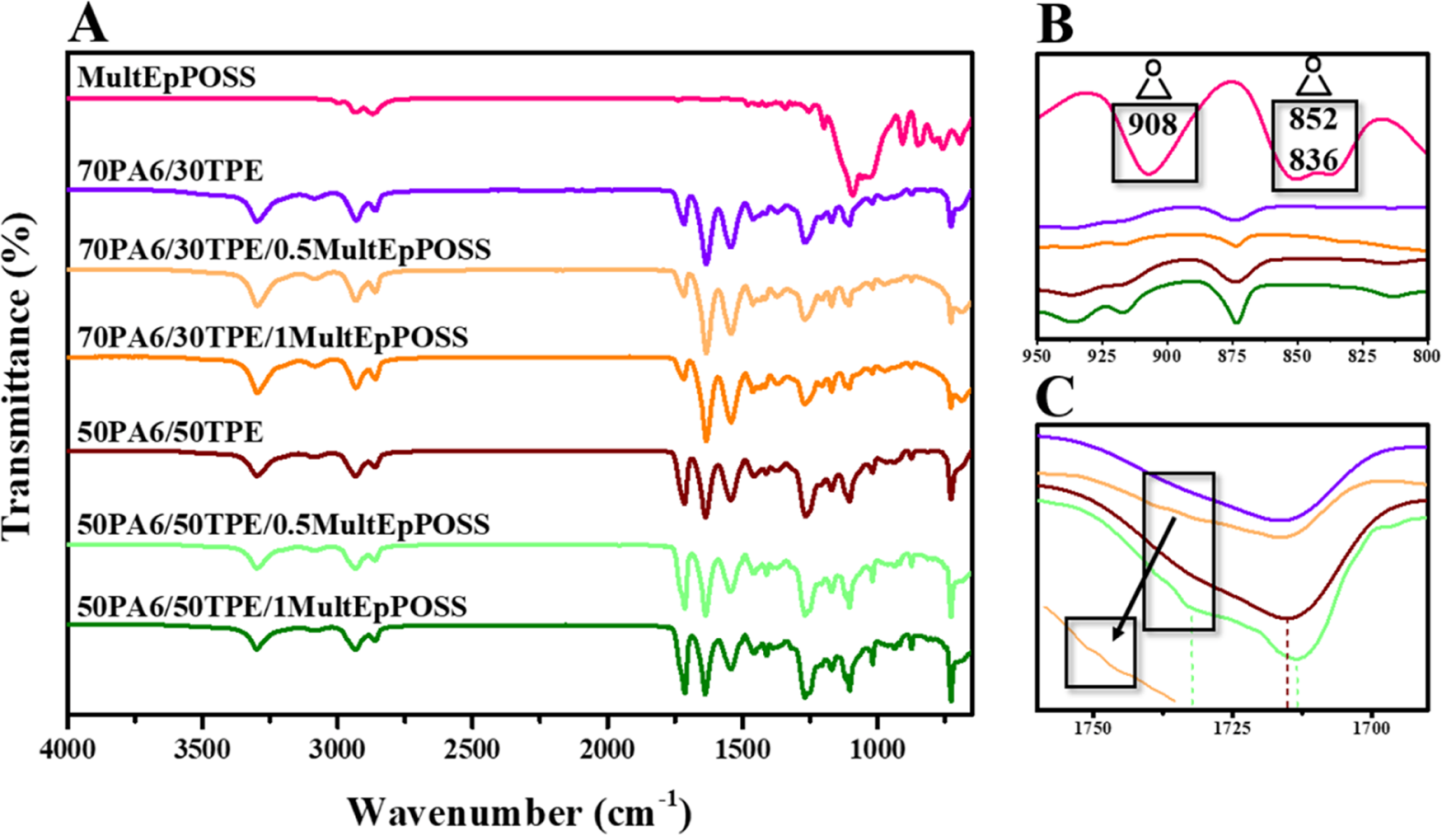
ATR-FTIR spectra of MultEpPOSS, PA6/TPE, and PA6/TPE/MultEpPOSS blends (A), the disappearance of epoxy peaks of naked MultEpPOSS in 70PA6/30TPE/1MultEpPOSS and 50PA6/50TPE/1MultEpPOSS blends (B), and the formation of tertiary amide peaks even at 0.5% MultEpPOSS content in PA6/TPE/MultEpPOSS blends (C).

**Scheme 1:**
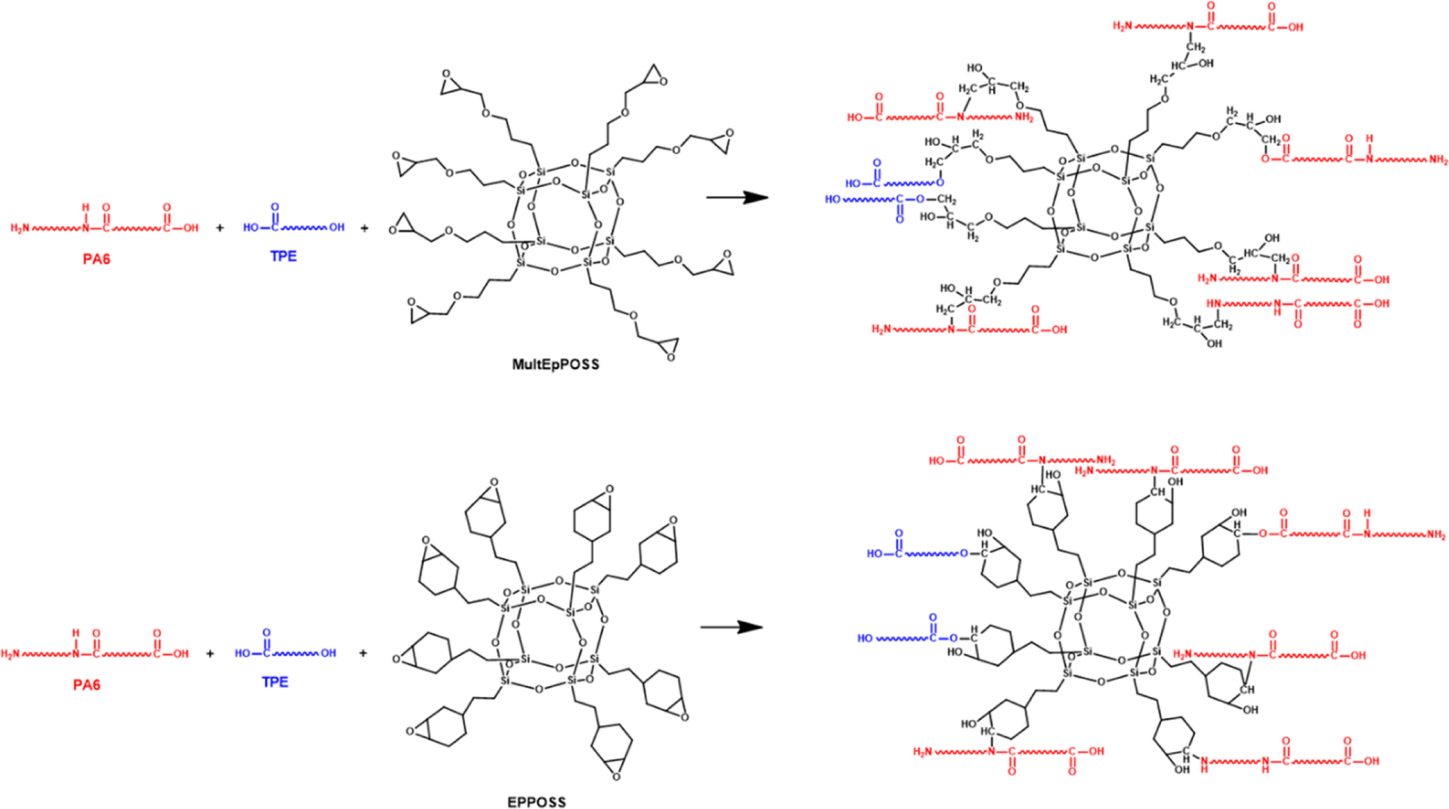
Possible Reactions between PA6, TPE, and MultEpPOSS/EPPOSS

The ATR-FTIR spectra of 70PA6/30TPE and 50PA6/50TPE blends with 0.5 and 1 wt % MultEpPOSS were compared with those without MultEpPOSS (Figure 2A). For instance, the disappearance of the vibrations of the epoxy groups of pure MultEpPOSS at approximately 908, 852, and 836 cm^–1^^40,41^ in all blends having 1% MultEpPOSS (Figure 2B) or 0.5% MultEpPOSS (data not shown in magnified form) is probably due to the reactions between highly reactive epoxy groups of POSS and proper groups in PA6/TPE, as indicated in Scheme 1. Also, a slight shift of the amide carbonyl group from 1715 cm^–1^ in the 50PA6/50TPE blend to 1713 cm^–1^ in the 50PA6/50TPE/0.5MultEpPOSS blend, the formation of a new distinctive peak at 1732 cm^–1^, and the peak broadening in this region in the ATR-FTIR spectra (Figure 2C) prove that tertiary amide groups are formed as a result of possible reactions between the epoxy groups of POSS and repeating amide groups in the main chain of PA6^40^ even at the existence of 0.5% MultEpPOSS (Figure 2C and Scheme 1). Similar behavior was observed in the 70PA6/30TPE/0.5MultEpPOSS blend as well (Figure 2C).

**Figure 3:**
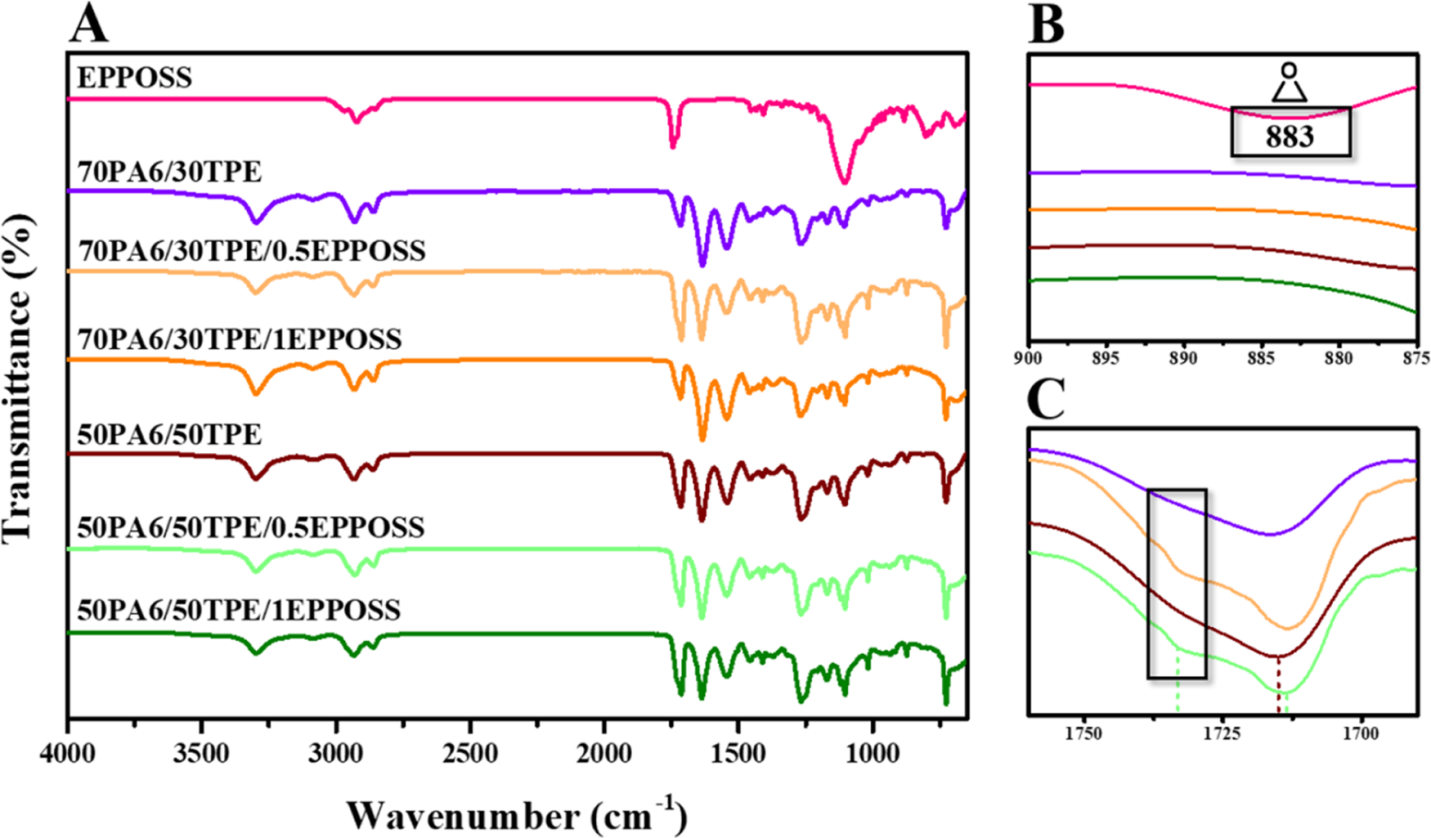
ATR-FTIR spectra of EPPOSS, PA6/TPE and PA6/TPE/EPPOSS blends (A), the disappearance of epoxy peaks of naked EPPOSS in 70PA6/30TPE/1EPPOSS and 50PA6/50TPE/1EPPOSS blends (B), and the formation of tertiary amide peaks even at 0.5% EPPOSS content in PA6/TPE/EPPOSS blends (C).

In the ATR-FTIR spectra of PA6/TPE/EPPOSS blends (Figure 3A), like PA6/TPE/MultEpPOSS blends as shown above in Figure 2, the vibrations of the epoxy groups of EPPOSS at approximately 883 cm^–1^^42^ disappeared in all blends containing 1% EPPOSS (Figure 3B) or 0.5% EPPOSS (data not shown in magnified form). Also, the carbonyl group of amides at 1715 cm^–1^ in PA6/TPE blends shifted to 1713 cm^–1^ in blends containing even 0.5% EPPOSS, and new peak formations occurred at 1732 cm^–1^ along with peak broadening (Figure 3C).

The possible reactions of the proper groups of PA6 and TPE with the epoxy groups of MultEpPOSS/EPPOSS are presented in Scheme 1. These reactions are (i) epoxy–amide reactions^40^ between the epoxy groups of POSS and repeating amide groups in the main chain of PA6, (ii) epoxy–amine and epoxy–acid reactions^43,44^ between highly reactive epoxy groups of MultEpPOSS/EPPOSS and amine and acid end groups of PA6, and (iii) epoxy–alcohol and epoxy–acid reactions^35,45,46^ between epoxy groups of MultEpPOSS/EPPOSS and alcohol and acid end groups of TPE. All reactions indicated above potentially influence the phase morphologies and characteristics of the PA6/TPE blends.

**Scheme 2:**
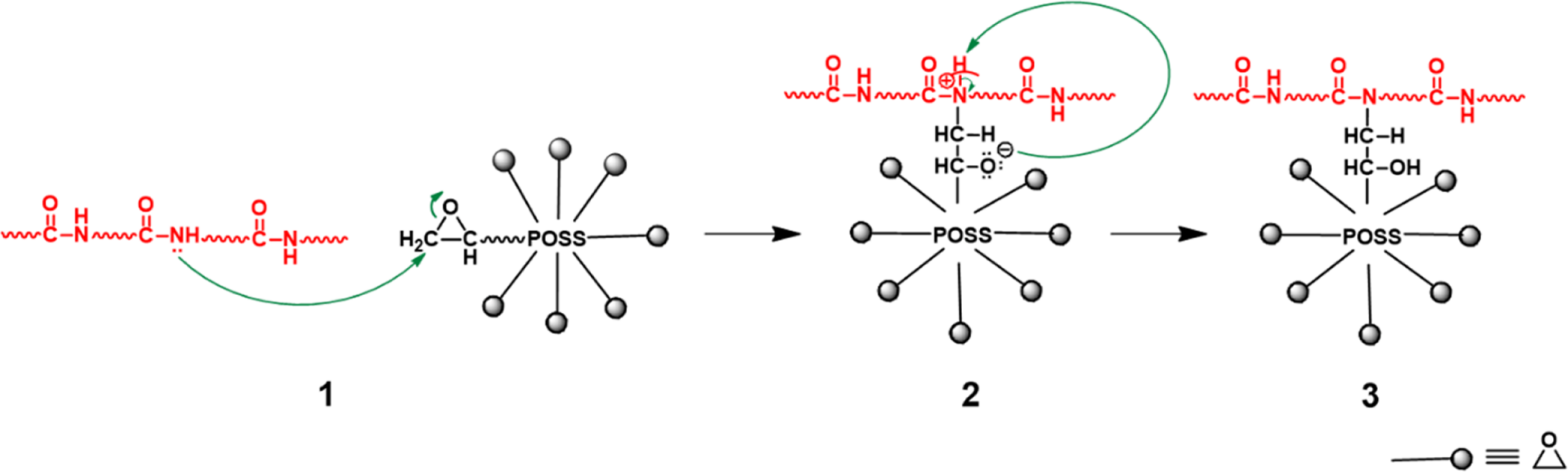
Epoxy–Amide Reaction Mechanism between Epoxy Groups of MultEpPOSS and Repeating Amide Groups of PA6

The epoxy–amide reactions between the epoxy groups of POSS and the repeating amide groups in the PA6 chains were confirmed by the formation of tertiary amide groups shown by ATR-FTIR (Figures 2C and 3C) analyses. On the other hand, possible epoxy–amine and epoxy–acid reactions between the epoxy groups of POSS and both the amine and acid end groups of PA6 and possible epoxy–alcohol and epoxy–acid reactions between the epoxy groups of POSS and both the alcohol and acid end groups of TPE could not be detected by ATR-FTIR analysis due to the existence of only a single end group in a long PA6 chain, as expected. The general mechanism of this reaction is that the lone pair of electrons on the nitrogen of the amide group in the PA6 repeating unit attacks the electrophilic methylene carbon next to the epoxide oxygen (1), resulting in intermediate (2) with a negative charge on the oxygen atom and a positive charge on the nitrogen atom. Then, the negatively charged oxygen atom takes a hydrogen atom from the positively charged nitrogen atom in intermediate (2), leading to the formation of tertiary amide (3) on the PA6 repeating main chain^40^ (Scheme 2). With the formation of the tertiary amide, the hydrogen bonding between PA6 chains decreases, and the symmetry of the chains is disrupted.

### 3.2 Selective Localization Measurements

The morphology of nanoparticle-reinforced polymer blends is influenced by the specific localization of the nanoparticles within the polymer blend. This selective localization behavior results from changes in the affinity between the nanoparticles and the two polymer components. The relationship between surface properties and nanoparticle localization can be characterized by the “wetting coefficient (ω_a_)” as given in eq 1
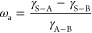
1where γ_S–A_ and γ_S–B_ refer to the interfacial tension between the nanoparticle and polymers A and B, respectively. Relying on the value of ω_a_, it is possible to provide information about nanoparticle localization. Thus, if ω_a_ > 1, it indicates that the nanoparticle is preferentially localized within polymer B, while if ω_a_ < −1, the nanoparticle tends to be located within polymer A. −1 < ω_a_ < 1 indicates that the nanoparticle is located at the interface between polymers A and B.

The interfacial energy between the two components was estimated by calculating the surface energies and their polar (AB) and dispersive (LW) components using eq 2
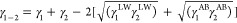
2The surface energy values of PA6 and TPE were obtained by calculating the contributions of basic (γ^–^), acidic (γ^+^), polar (γ^AB^), and dispersive (γ^LW^) components, as well as the contact angle (θ) data, using eq 3.
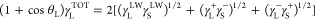
3The MultEpPOSS and EPPOSS nanoparticles are in liquid form at room temperature. Therefore, the surface energies of POSSs were determined by drop casting on known probe solids (Table 3). The surface energies of polymers and POSSs were then estimated by using eqs 2 and 3, respectively, and the results are reported in Table 4. The contact angles of the samples used to determine the surface energy values of the components can be found in the Supporting Information (Tables S1 and S2).

**Table 4:** Surface Energies (mJ/m^2^) of Neat Polymers and POSSs

sample	basic component (γ_S_^–^)	acidic component (γ_S_^+^)	acid–base component (γ_S_^AB^)	dispersive component (γ_S_^LW^)	total surface energy (γ_S_^TOT^)
PA6	11.0	0.1	0.4	43.3	43.7
TPE	17.8	0.3	4.7	46.8	51.5
MultEpPOSS	2.0	10.5	9.1	16.0	25.0
EPPOSS	28.0	20.6	48.0	26.5	74.5

Table 5 shows the interfacial energies and wetting coefficient values for polymer–POSS binary systems. The wetting coefficient values of MultEpPOSS and EPPOSS were determined as 1.5 and 6.7 mJ/m^2^, respectively, by using eq 1. The values of the wetting coefficient above 1 (ω_a_ > 1) suggest that both POSS nanoparticles are preferentially located in the TPE phase. This can be attributed to the chemical compatibility between polar MultEpPOSS and EPPOSS and the more hydrophilic TPE and facilitated by their high acidic–basic component (γ_S_^AB^).

**Table 5:** Interfacial Energies between Polymers–POSSs and the Wetting Coefficients of POSSs*

sample	MultEpPOSS	EPPOSS
γ_PA6-POSS_	12.3	41.6
γ_TPE-POSS_	8.8	25.6
ω_a_	1.5	6.7

* γ_PA6-TPE_ = 2.4 mJ/m^2^

### 3.3 Scanning Electron Microscopy (SEM)

**Figure 4:**
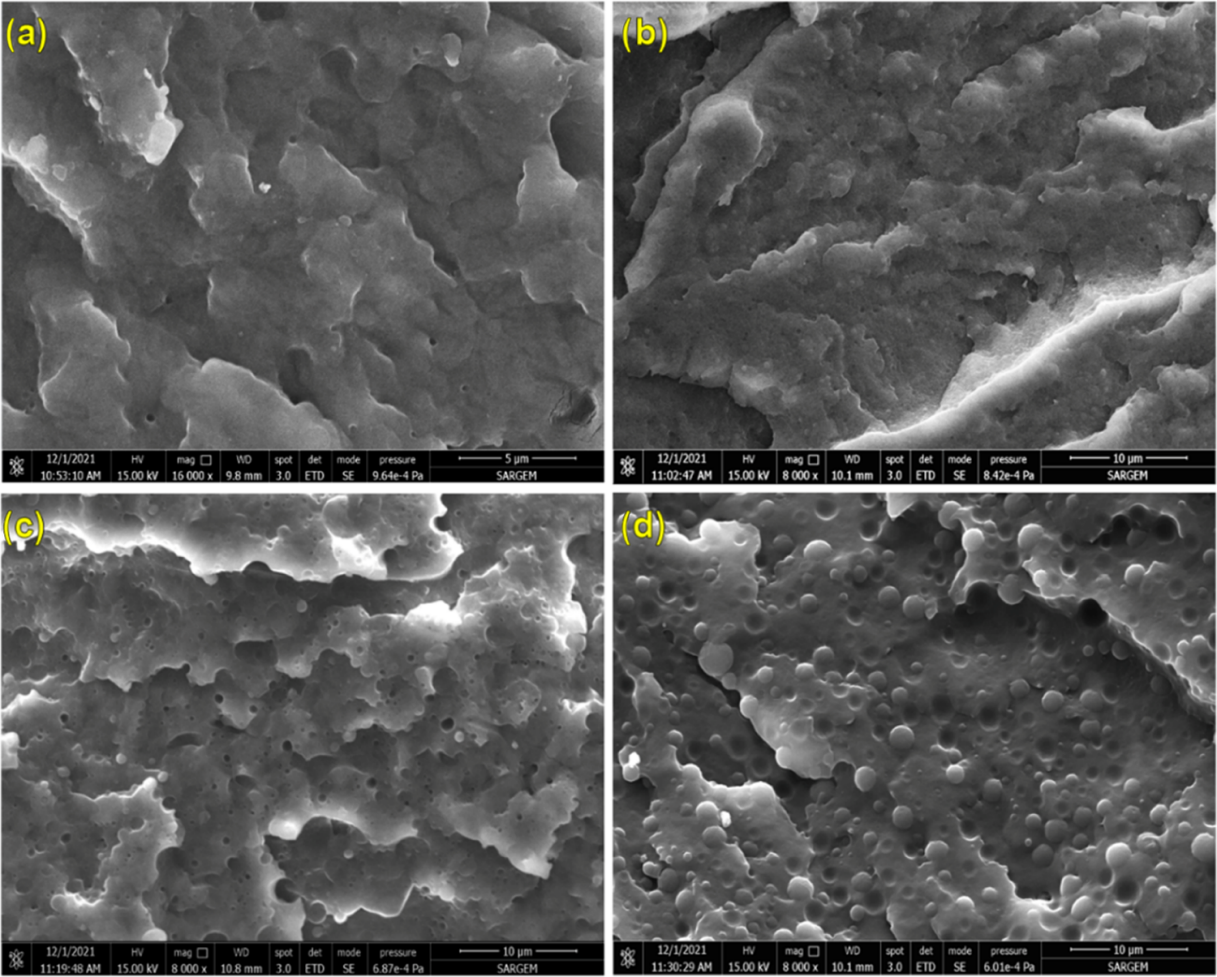
SEM images of (a) PA6, (b) TPE, (c) 70PA6/30TPE, and (d) 50PA6/50TPE (magnification: ×8000).

**Figure 5:**
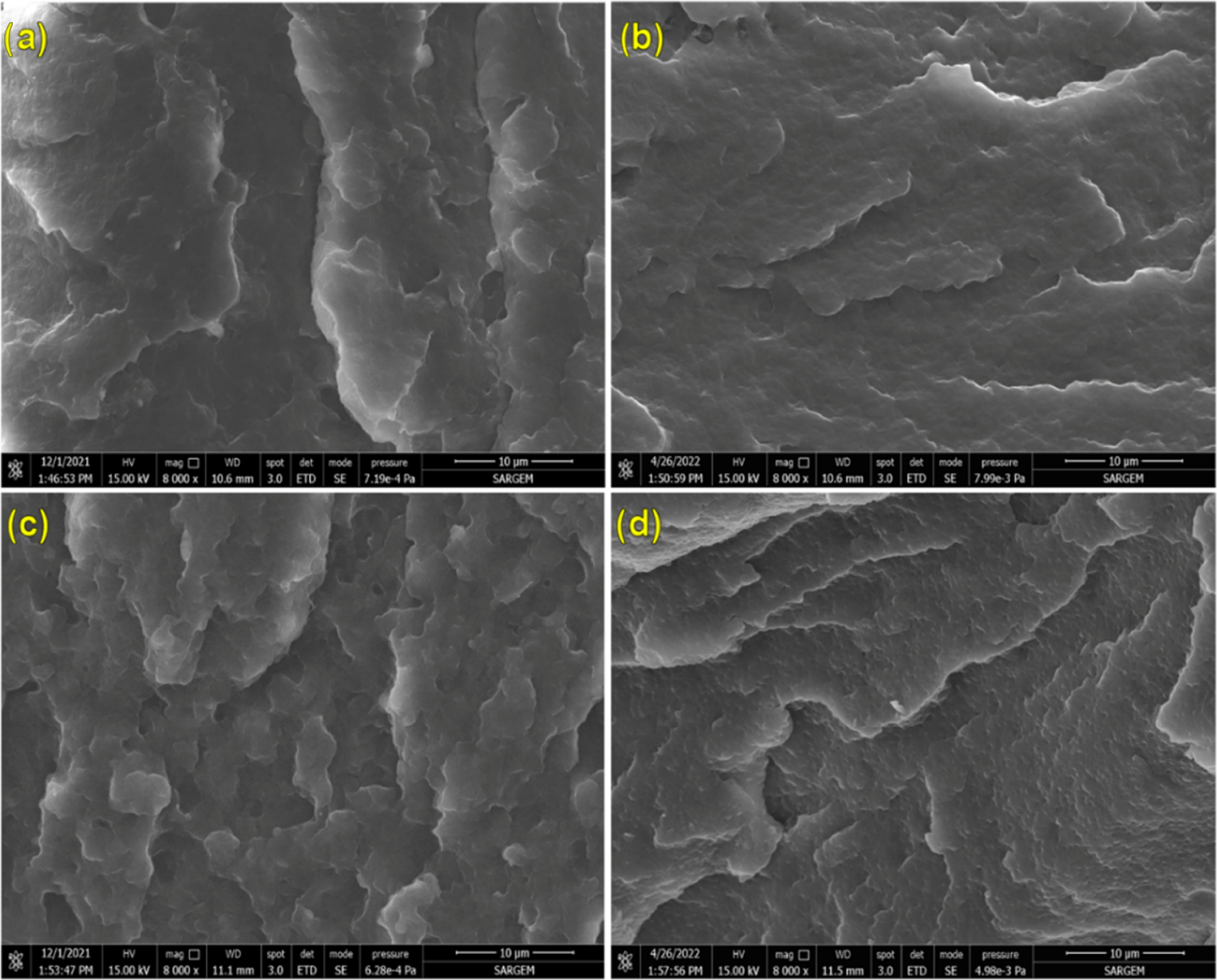
SEM images of (a) 70PA6/30TPE/0.5MultEpPOSS, (b) 70PA6/30TPE/1MultEpPOSS, (c) 50PA6/50TPE/0.5MultEpPOSS, and (d) 50PA6/50TPE/1MultEpPOSS (magnification: ×8000).

**Figure 6:**
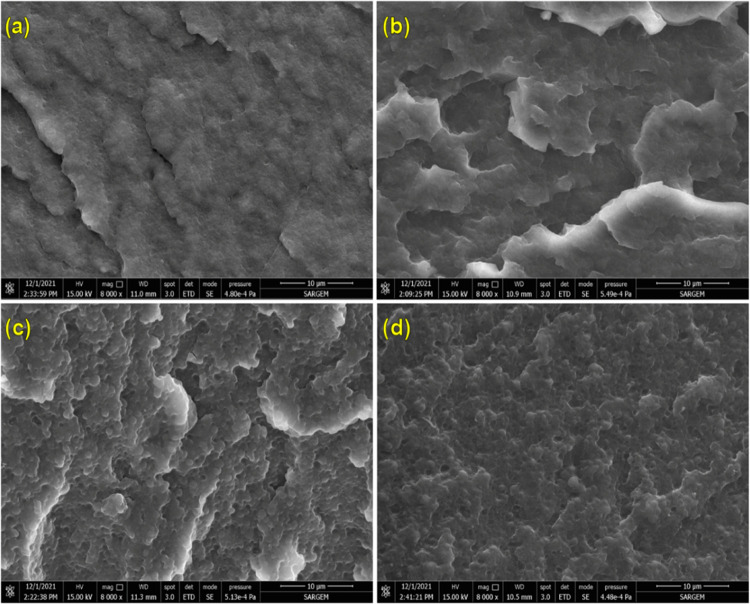
SEM images of (a) 70PA6/30TPE/0.5EPPOSS, (b) 70PA6/30TPE/1EPPOSS, (c) 50PA6/50TPE/0.5EPPOSS, and (d) 50PA6/50TPE/1EPPOSS (magnification: ×8000)

To achieve the desired properties in polymer blends, the dispersion of the dispersed phase in the matrix is extremely important. To improve the physical and chemical properties, it is crucial to reduce the size of the dispersed phase and ensure its homogeneous dispersion within the continuous phase. This is necessary for increased interfacial interaction between the components. SEM images of pure PA6, pure TPE, and PA6/TPE blends with and without POSS compatibilizers are presented in Figures 4–6, and the corresponding average particle sizes (*d*_AVG_) calculated from these images are summarized in Table 6. When the SEM images of pure PA6 and pure TPE are examined (Figure 4), distinctive features are revealed. The presence of parallel plateau-like structures on the surface of pure PA6 indicates a relatively brittle surface morphology. In contrast, the fracture surface of pure TPE exhibits shear bands, indicating plastic deformation. This behavior is attributed to the flexible poly(alkylene ether glycol) terephthalate segments in the TPE structure, which have a high energy absorption capacity. Regardless of the blend ratio, PA6/TPE blends exhibit a two-phase morphological structure (Figure 4C,D). It was observed that the average particle size of TPE increased with increasing TPE content in the blend (Table 6). These findings indicate that as the TPE content increases, PA6/TPE blends shift from partial miscibility to thermodynamic immiscibility, leading to poor interfacial interaction.

**Table 6:** Dispersed Phase Particle Sizes Obtained from SEM Images

sample	*d*_AVG_ (μm)
70PA6/30TPE	0.61 ± 0.2
70PA6/30TPE/0.5MultEpPOSS	
70PA6/30TPE/1MultEpPOSS	
70PA6/30TPE/0.5EPPOSS	0.40 ± 0.1
70PA6/30TPE/1EPPOSS	0.26 ± 0.1
50PA6/50TPE	1.39 ± 0.3
50PA6/50TPE/0.5MultEpPOSS	0.46 ± 0.1
50PA6/50TPE/1MultEpPOSS	0.43 ± 0.1
50PA6/50TPE/0.5EPPOSS	0.59 ± 0.1
50PA6/50TPE/1EPPOSS	0.41 ± 0.1

As shown in Figures 5 and 6 and summarized in Table 6, the addition of MultEpPOSS and EPPOSS to PA6/TPE blends led to a significant reduction in the average particle size of TPE, regardless of the PA6/TPE blend ratio. In the case of the 70PA6/30TPE blend, where PA6 forms the continuous phase, the addition of MultEpPOSS and EPPOSS resulted in the formation of an almost single phase and highly stable phase morphology. This is particularly evident in the 70PA6/30TPE blend containing MultEpPOSS, where the dispersed phase of TPE is not observed. In 50PA6/50TPE blends containing both MultEpPOSS and EPPOSS, TPE particles are homogeneously dispersed in the matrix, as observed from SEM images. The compatibilization efficiency of MultEpPOSS and EPPOSS is remarkable at both low and high loading levels, especially in 70PA6/30TPE blends with a higher PA6 content. It was also observed that the phase morphologies of 50PA6/50TPE blends were significantly improved in the presence of MultEpPOSS and EPPOSS. The achievement of highly stable phase morphologies in PA6/TPE blends in the presence of POSS nanoparticles can be attributed to the following factors: (1) The multiple epoxy functional groups of MultEpPOSS and EPPOSS react with –NH_2_ and –COOH groups at the chain end of PA6, as well as with –NHCO groups in the repeating unit of PA6 and/or –COOH and –OH groups at the chain end of TPE, resulting in the formation of high-molecular-weight PA6-*co*-POSS-*co*-TPE block and/or graft copolymers (Scheme 1). (2) As shown by the selective localization test results, MultEpPOSS and EPPOSS nanoparticles were positioned in the TPE phase, reducing the viscoelastic differences of the two matrices and contributing to droplet breakage, especially in 50PA6/50TPE blends.

### 3.4 Rheological Analyses

**Figure 7:**
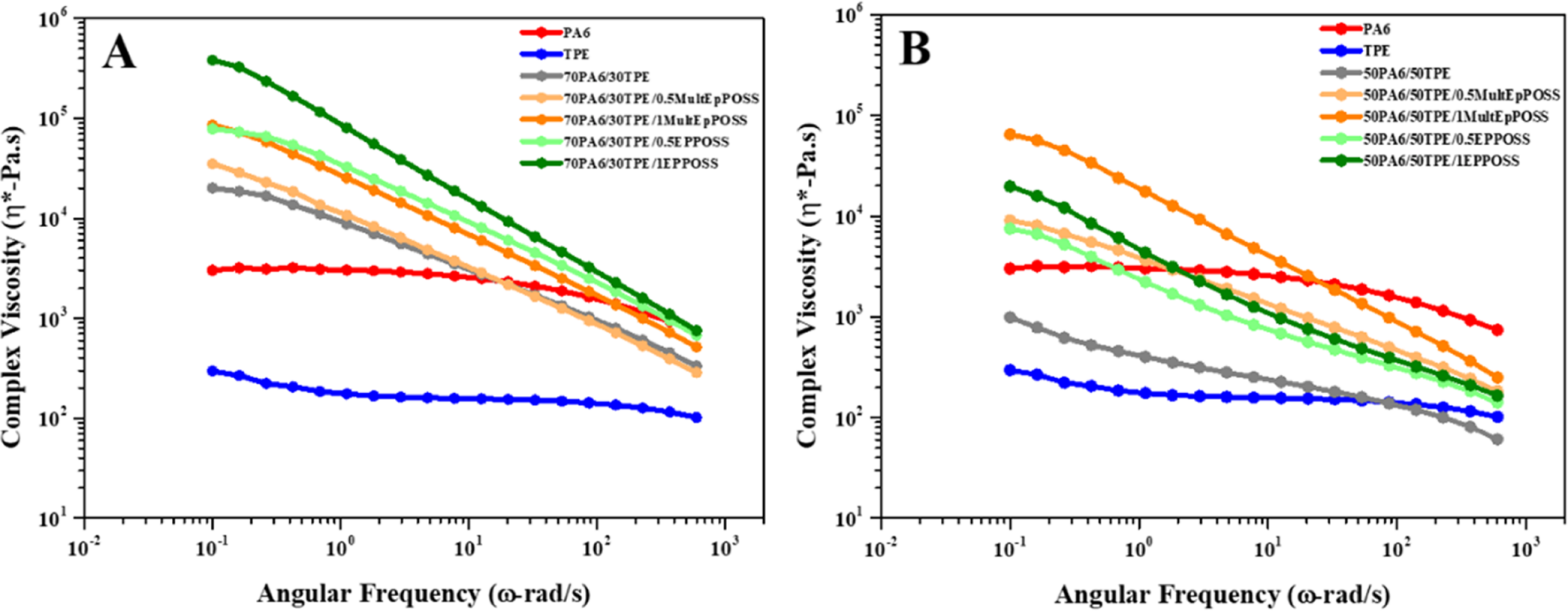
Complex viscosity versus angular frequency of (A) 70PA6/30TPE and 70PA6/30TPE/POSS blends and (B) 50PA6/50TPE and 50PA6/50TPE/POSS blends.

The dynamic rheological behavior of polymers is strongly affected by changes in their molecular structure. Hence, the interaction between polymers under certain conditions can be evaluated by studying their rheological properties.^47^Figure 7 presents the complex viscosity changes as a function of the angular frequency of pure polymers, PA6/TPE blends with and without POSS. The complex viscosity of pure PA6 displays higher values compared to pure TPE over the whole frequency range. This can be explained by stronger secondary interactions of PA6, such as hydrogen bonding. Additionally, pure PA6 and pure TPE exhibit Newtonian flow behavior in the measured frequency range independent of the frequency, indicating typical unentangled polymer melt.^48,49^

At the low-frequency region, the complex viscosity of the 70PA6/30TPE blend exceeds that of pure PA6. This can be attributed to the formation of hydrogen bonds between the N–H proton of PA6 and the carbonyl (C=O) group of TPE. As a result, higher complex viscosity values are observed, indicating the degree of partial compatibility obtained through in situ copolymer formation in the blends.^50^ Furthermore, it was observed that the viscosity of the blend was lower than the viscosity of PA6 due to the decrease in the interaction between PA6 and TPE with increasing TPE ratio in PA6/TPE blends. This can be attributed to the nucleating agent effect of PA6 for the TPE phase in the 50PA6/50TPE blend, which triggered the phase separation of the components, as discussed in the DSC test results. Notably, at low frequencies, a more Newtonian flow behavior was observed instead of a shear thinning behavior. This finding can be interpreted as an indication that thermodynamic incompatibility between the components intensifies in the case of a co-continuous phase morphology. These findings are also consistent with the SEM analysis results. However, as the angular frequency increases, it is observed that the viscosity values of all blends exhibit a sudden decrease. This can be attributed to the relaxation of polymer chains within the blends.^51^

At any considered angular frequency, the PA6/TPE samples containing MultEpPOSS and EPPOSS exhibit higher complex viscosity values compared with the PA6/TPE samples without a compatibilizer. This enhancement in complex viscosity can be attributed to the presence of POSS molecules, which contain multiple epoxy groups and facilitate increased interactions between the components. These interactions may arise from possible reactions between carboxylic acid (−COOH) and hydroxyl (−OH) groups of TPE and the amide (−NHCO−), amine (−NH_2_), and/or carboxylic acid (−COOH) groups of PA6 as discussed earlier. As a result, graft and block copolymers form at the interface, resulting in improved complex viscosity values. Furthermore, the addition of POSS intensifies the shear thinning behavior observed with an increasing angular frequency in the samples. This can be attributed to the introduction of long-chain branching, which resulted in more entanglements and higher molecular weights through the addition of POSS during the reactive blending process, leading to improved melt stability of the polymer.^45,52,53^ In addition to these, as can be seen from the SEM results, the significant decrease in the dispersed phase particle size with the addition of POSS, the increase in the interaction between the phases and thus the interphase becoming more intense, resulted in significant improvements in the complex viscosity.^45,54^

In the presence of POSS molecules, the 70PA6/30TPE blend exhibits higher complex viscosity values across the entire frequency range compared with the POSS-compatibilized 50PA6/50TPE blend. Moreover, the complex viscosity curves display a steeper slope, indicating the formation of graft or block copolymers. As the proportion of PA6 decreases in the blend, the degree of long-chain branching decreases, leading to lower complex viscosity values. Additionally, the higher complex viscosity value observed in the 50PA6/50TPE blend containing 1 wt % MultEpPOSS can be attributed to the higher reactivity of MultEpPOSS, likely caused by steric hindrance resulting from the cycloaliphatic structure of EPPOSS.

**Figure 8:**
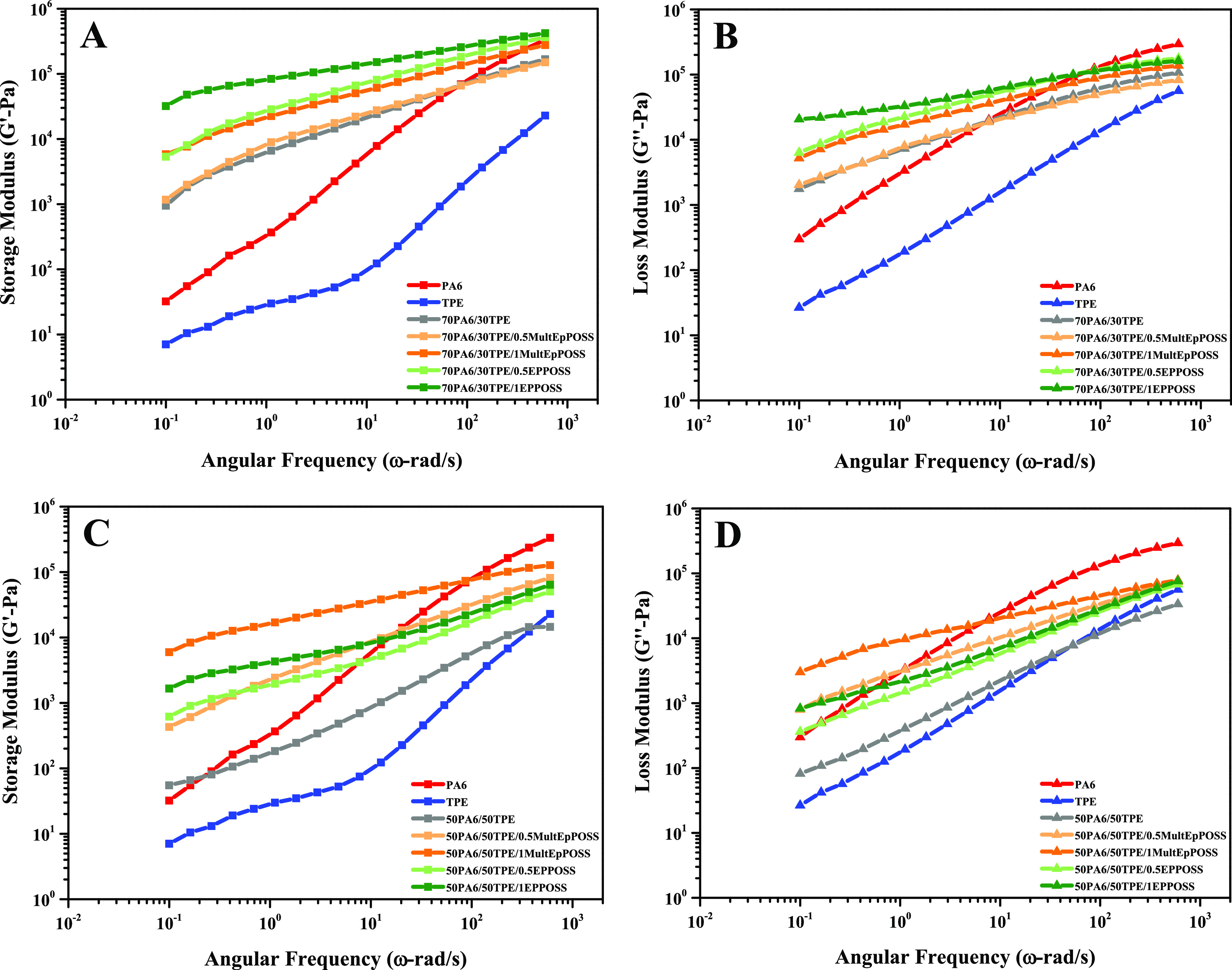
Modulus versus angular frequency: (A) storage modulus of 70PA6/30TPE and 70PA6/30TPE/POSS blends, (B) loss modulus of 70PA6/30TPE and 70PA6/30TPE/POSS blends, (C) storage modulus of 50PA6/50TPE and 50PA6/50TPE/POSS blends, and (D) loss modulus of 50PA6/50TPE and 50PA6/50TPE/POSS blends.

Figure 8 presents the storage and loss modulus changes of the samples, depending on the frequency. In the case of both pure polymers, the loss modulus consistently exhibits higher values compared to the storage modulus, indicating the dominance of viscous (liquid-like) properties in the polymers. In the low-frequency range, the 70PA6/30TPE blend displays higher storage and loss modulus values than pure PA6, primarily attributed to higher hydrogen bond formation, as mentioned previously. In cases where the continuous phase is PA6, it is observed that the loss modulus is initially high and subsequently intersects with the storage modulus at high frequencies, resulting in lower values. These observations suggest that molecular forces increase under shear stress, causing the PA6/TPE blends to behave as elastic solids at high frequencies.^55^ Conversely, in the 50PA6/50TPE blend, the loss modulus and storage modulus exhibit intermediate values compared with the pure polymers across the entire frequency range. Additionally, in the 50PA6/50TPE blend, the loss modulus surpasses the storage modulus across the entire frequency range. This suggests a more liquid-like behavior in the blend and indicates a tendency toward phase separation as the proportion of PA6 decreases.

The increments in the storage and loss modulus values of PA6/TPE blends with the addition of POSS can be attributed to the primary interactions occurring between the multiple epoxy groups in the structures of the POSSs and the reactive groups present in PA6 and TPE. As a result, the formation of block and/or graft copolymers at the interphase reduces the interfacial tension between PA6 and TPE, leading to notable enhancements in the rheological properties of the blends. The rheological analysis results revealed that EPPOSS demonstrated superior rheological properties in the blend comprising 30% TPE, while MultEpPOSS exhibited higher rheological properties in the blend with a co-continuous phase morphology. Moreover, this trend became more pronounced as the loading rate of POSS increased. The observed phenomenon can be attributed to the formation of steric hindrance by the cycloaliphatic epoxy group attached to the lattice structure of EPPOSS as the TPE ratio increases. Nevertheless, it is noteworthy that the rheological properties were significantly enhanced, independent of the type of POSS molecules.

### 3.5 Tensile Test

**Figure 9:**
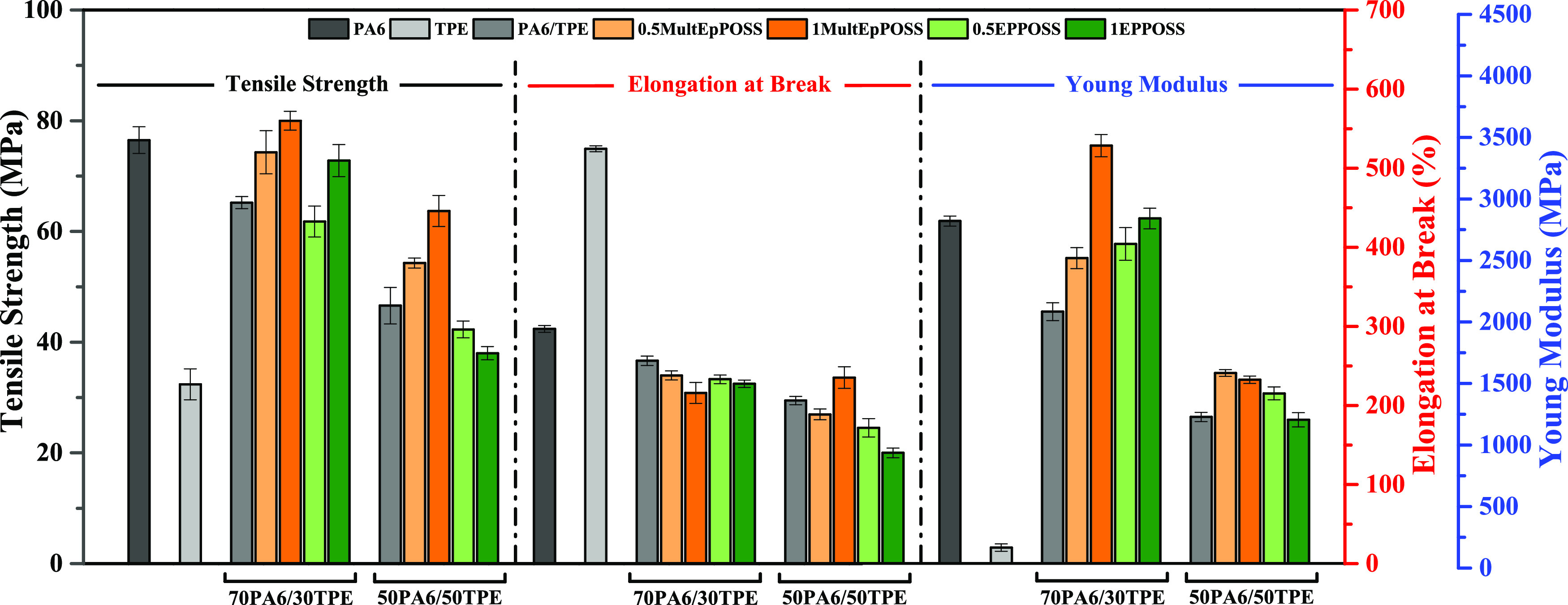
Tensile test results of neat polymers and PA6/TPE and PA6/TPE/POSS blends.

Tensile tests were performed to evaluate the mechanical properties of the samples. The stress–strain curves obtained from the tensile tests were used to analyze the variations in the tensile strength, elongation at break, and Young’s modulus. Figure 9 illustrates the alterations in the mechanical properties of the pure polymers as well as the PA6/TPE blends with and without POSS compatibilizers.

The addition of TPE to pure PA6 results in a notable reduction in the mechanical properties, which is attributed to the insufficient interfacial interaction between the constituents of the polymer blend and the formation of an unstable morphology. This decrease in mechanical properties is much more prominent as the TPE content increases in PA6/TPE blends. These observations are in agreement with the findings obtained from the SEM analysis. Moreover, the incorporation of TPE into PA6 leads to a decrease in interphase interaction and an increase in thermodynamic instability within the PA6/TPE blends, which aligns with the outcomes of the rheology analysis.

The mechanical behavior of particle-reinforced composite materials is influenced by various factors, including the interfacial interaction between the components, particle geometry, particle content in the matrix, and particle size.^56^ At the same time, in incompatible polymer blends, the mechanical properties are closely related to the phase morphology.^57^ The addition of MultEpPOSS to 70PA6/30TPE and 50PA6/50TPE blends resulted in a notable enhancement in the tensile strength values. Furthermore, the tensile strength of PA6/TPE blends exhibited a continuous improvement as the loading ratio of MultEpPOSS increased (Figure 9). For EPPOSS-compatibilized PA6/TPE blends, the improvement of tensile strength of PA6/TPE blends was achieved for the 70PA6/30TPE blend at higher EPPOSS concentrations. On the other hand, the tensile strength of 50PA6/50TPE decreased in the presence of EPPOSS. However, it was found that MultEpPOSS-compatibilized blends showed higher tensile strength values in comparison with EPPOSS-compatibilized blends. This can be attributed to the higher reactivity of MultEpPOSS toward the PA6/TPE blend, especially with the PA6 phase. These observations for tensile strength values can be attributed to the enhanced interfacial interaction achieved between the immiscible components, PA6 and TPE, through reactive blending in the presence of POSS nanoparticles, as explained in the SEM analysis. The findings from the tensile test indicate that EPPOSS, similar to MultEpPOSS, functions as an effective compatibilizer via their epoxy groups for the polymer blends, especially with a continuous PA6 phase. POSS nanoparticles act as emulsifiers, reducing the interfacial tension between PA6 and TPE through primary strong interactions, thereby enabling the attainment of a stable phase morphology. Consequently, the notable improvements in tensile strength, particularly in PA6/TPE blends with a higher PA6 content, can be attributed to the increased interfacial interactions.

The incorporation of both MultEpPOSS and EPPOSS into the 70PA6/30TPE and 50PA6/50TPE blends resulted in enhancements in Young’s modulus values. This can be attributed to the positioning of MultEpPOSS and EPPOSS within the TPE phase, resulting in particle breakage and dispersion as smaller particles within the matrix. As shown in Table 6, POSS incorporation significantly decreased the average particle size of the dispersed phase. As a consequence, a larger interfacial area between PA6 and TPE was formed, leading to enhanced stiffness properties of the material.

In general, a reduction in the elongation at break values of the samples was observed in the presence of POSS nanoparticles (Figure 9). This decrease in elongation at break values can be attributed to the rigid cage structure of POSS and the increase in chain entanglement density induced by the presence of POSS, independent of the POSS type. Specifically, in the presence of MultEpPOSS and EPPOSS, it is proposed that the rigid segments derived from the POSS cage structure of the graft or block copolymers formed at the PA6-TPE interface impede plastic deformation, resulting in lower elongation at break values. In a general conclusion, the mechanical analysis test results indicate that MultEpPOSS exhibits higher reactivity compared to EPPOSS.

### 3.6 Impact Test

**Figure 10:**
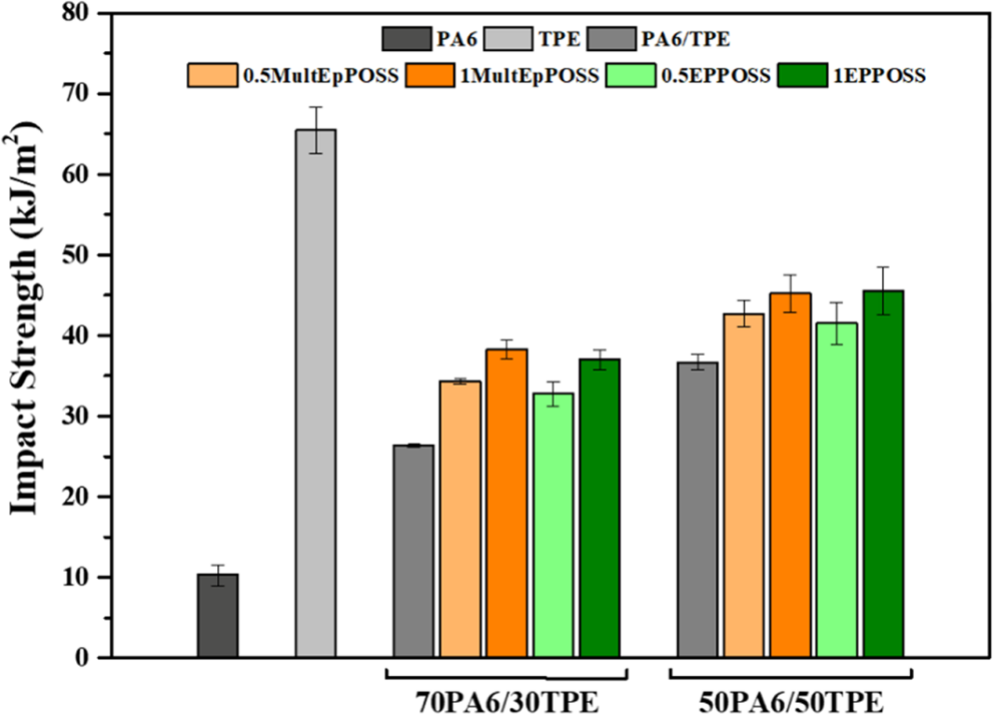
Izod impact test results of neat polymers and PA6/TPE and PA6/TPE/POSS blends.

To assess the impact strength of the samples prepared in this study, Izod impact strength tests were conducted on samples featuring 2 mm V-shaped notches. The Izod impact strength values of the PA6/TPE blends are presented in Figure 10. The 10 kJ/m^2^ impact strength of pure PA6 increased upon the addition of TPE. For instance, the incorporation of 50% TPE into PA6 resulted in a notable 256% increase in the Izod impact strength. Pure PA6 exhibits a relatively high glass transition temperature of approximately 47 °C, which accounts for its relatively low impact strength of around 10 kJ/m^2^. In contrast, pure TPE possesses a low glass transition temperature of approximately −55 °C due to its elastomeric structure, rendering it highly elastic and tough. Consequently, the addition of TPE with exceptional impact strength to PA6 led to significant improvements in the Izod impact strength values within the PA6/TPE blends.

The incorporation of POSS nanoparticles containing multiple epoxy groups into the PA6/TPE blends resulted in notable enhancements in the Izod impact strength, independent of the type of POSS employed. The impact strength improvement was particularly prominent in blends containing MultEpPOSS. Numerous studies in the literature have highlighted that the impact strength of polymer blends can be enhanced through reactive compatibilization, leading to increased interfacial interactions.^58^ The impact strength of polymer blends is greatly influenced by the size and distribution of the dispersed phase within the matrix. The presence of small and homogeneously dispersed particles in the matrix contributes to a higher toughness for several reasons. First, the localized stress concentration caused by particle cavitation results in plastic deformation of the matrix, altering the stress distribution surrounding the dispersed phase.^59,60^ However, it is crucial to ensure that cavitated particles do not initiate fracture processes; hence, these particles should remain very small in size and not reach dimensions that can induce crack formation.^61^ Therefore, regardless of the specific PA6/TPE blend, the addition of POSS molecules containing multiple epoxy groups contributes to increased molecular mass, a reduction of dispersed phase particle sizes depending on the matrix composition, and the formation of block and/or graft copolymers among the polymer components. Consequently, this leads to substantial improvement in the Izod impact strength values.

### 3.7 Differential Scanning Calorimetry (DSC) Analyses

**Figure 11:**
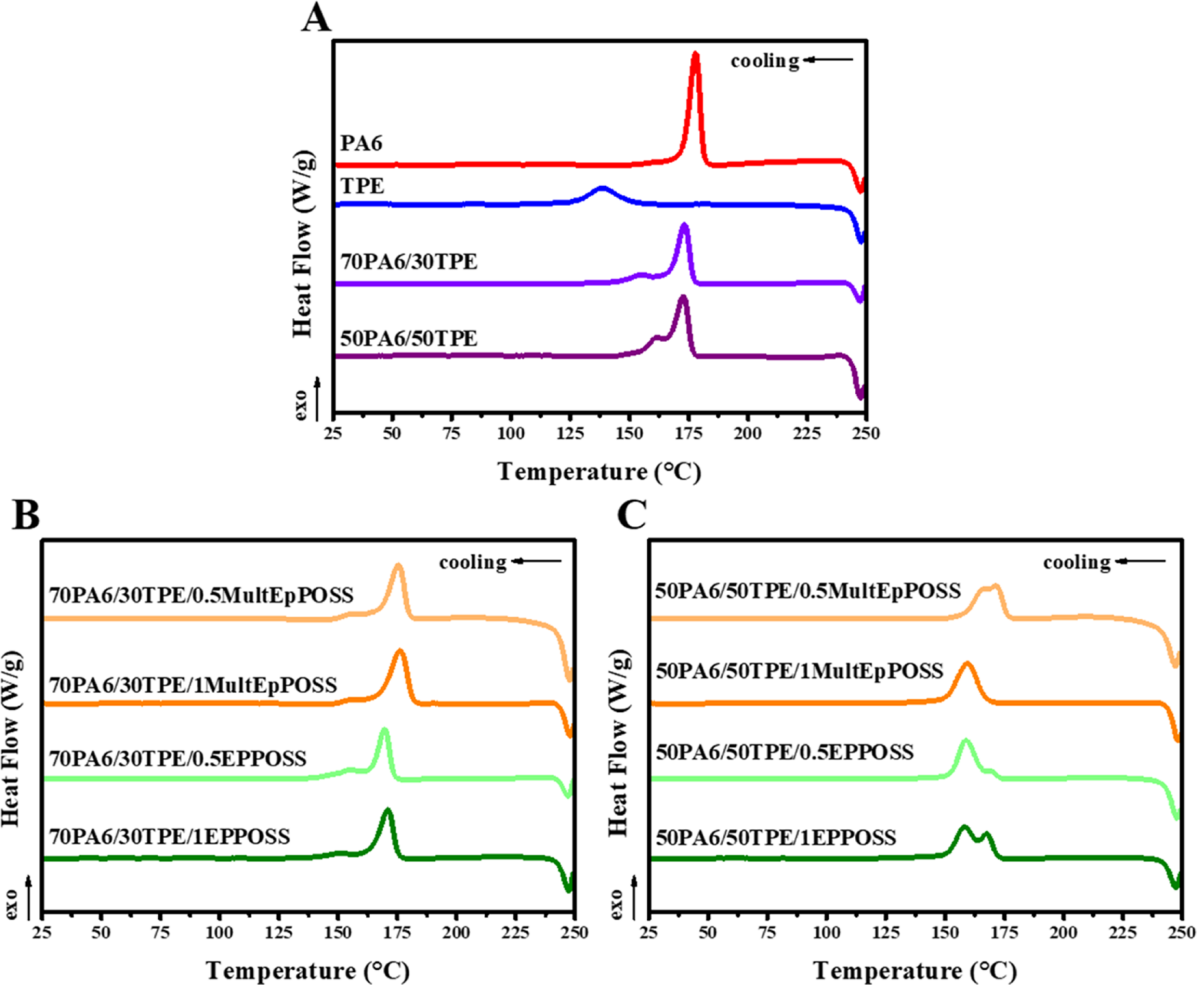
Cooling thermograms of (A) neat polymers and PA6/TPE blends (B) 70PA6/30TPE/POSS blends, and (C) 50PA6/50TPE/POSS blends.

**Figure 12:**
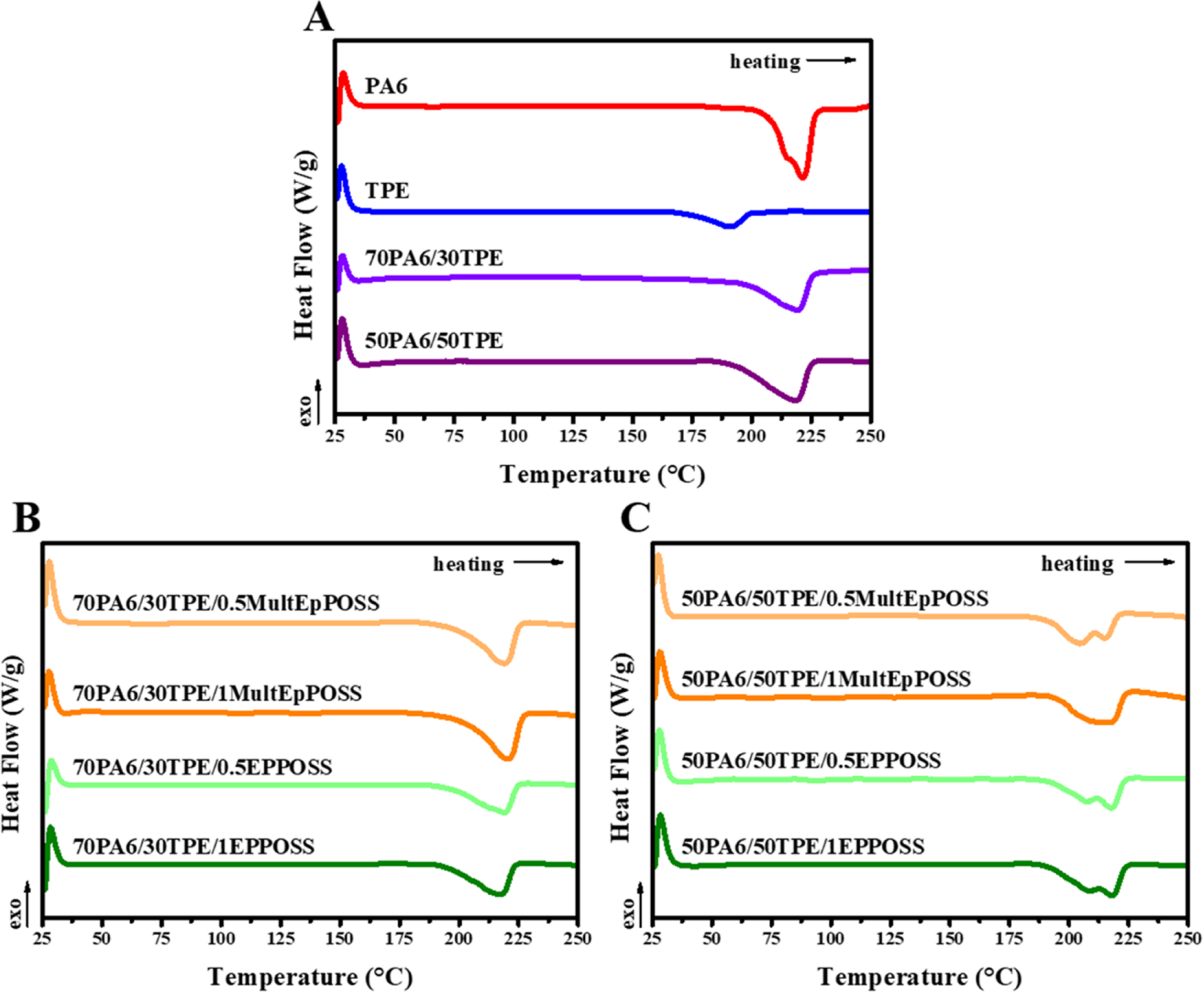
Second heating thermograms of (A) neat polymers and PA6/TPE blends, (B) 70PA6/30TPE/POSS blends, and (C) 50PA6/50TPE/POSS blends.

The thermal transitions of pure PA6, pure TPE, and PA6/TPE blends with and without a compatibilizer were examined using DSC analyses. The cooling and second heating thermograms are given in Figures 11 and 12, respectively. The results obtained from DSC analyses are summarized in Table 7.

**Table 7:** Thermal Properties of Neat Polymers, PA6/TPE, and PA6/TPE/POSS Blends

	cooling			second heating	
sample	*T*_c,onset_ (°C)	*T*_c,endset_ (°C)	Δ*H*_c_ (J/g)	*T*_m_ (°C)	Δ*H*_m_ (J/g)
PA6	183.9	169.2	55.5	221.4	48.6
TPE	154.6	124.8	23.1	190.8	12.8
					
70PA6/30TPE	179.3	145.8	41.7	219.2	39.8
70PA6/30TPE/0.5MultEpPOSS	182.5	149.9	41.6	219.0	37.2
70PA6/30TPE/1MultEpPOSS	183.5	148.5	52.3	220.2	42.2
70PA6/30TPE/0.5EPPOSS	175.8	143.9	35.6	218.9	29.8
70PA6/30TPE/1EPPOSS	177.9	144.2	36.7	217.2	37.1
					
50PA6/50TPE	178.7	145.7	52.9	218.2	49.2
50PA6/50TPE/0.5MultEpPOSS	178.0	150.0	37.9	204.3–215.8	26.1
50PA6/50TPE/1MultEpPOSS	169.9	142.2	35.2	217.0	29.5
50PA6/50TPE/0.5EPPOSS	174.5	147.7	31.8	206.6–218.4	25.1
50PA6/50TPE/1EPPOSS	173.1	148.1	36.8	207.3–218.8	30.2

Pure PA6 exhibits a narrower temperature range for crystallization compared to pure TPE, which exhibits a broader crystallization range (Figure 11 and Table 7). The onset crystallization temperature (*T*_c,onset_) of pure TPE is 154.6 °C, which indicates a slower crystallization process compared to PA6. The higher enthalpy of melt crystallization (Δ*H*_c_) value of PA6 indicates the existence of larger crystal sizes. When 30 wt % TPE was added to PA6, separate crystallization behaviors were observed in the molten phase with a similar trend in the 50PA6/50TPE blend, which indicates immiscible crystallization behavior. Furthermore, the onset crystallization temperature of TPE in PA6/TPE blends shifts to higher values compared to pure TPE. This shows that PA6 acts as a nucleating agent for TPE, and with a 50 wt % TPE ratio, the crystallization of TPE starts when the crystallization stage of PA6 is almost complete. When MultEpPOSS is added to the 70PA6/30TPE blend, the onset crystallization temperature of the PA6 phase increases by about 4 °C from 179.3 to 183.5 °C. The presence of MultEpPOSS also significantly decreases the average particle size of the dispersed TPE phase, providing nucleation sites for the PA6 phase and shifting the onset crystallization temperature values to higher temperatures. The higher Δ*H*_c_ values obtained in the presence of MultEpPOSS suggest that the nucleation density in the blend is increased. In the presence of EPPOSS, a two-phase crystallization behavior similar to the blend without POSS was observed, but a significant decrease in melt crystallization enthalpy values was recorded. For instance, the Δ*H*_c_ value of the 70PA6/30TPE blend, which was initially 41.7 J/g, decreases to 36.7 J/g with the addition of 1 wt % EPPOSS. This reduction is due to the suppression of crystallization of the components via the formation of graft and/or block copolymers in the presence of EPPOSS. With the addition of MultEpPOSS to the 50PA6/50TPE blend, important changes in the crystallization behavior of the components were observed. At a concentration of 0.5 wt % MultEpPOSS, an almost single-phase crystallization behavior is observed, and increasing the MultEpPOSS concentration to 1 wt % results in a single crystallization exotherm. The essential condition for cocrystallization is that both PA6 and TPE must be present at the crystal growth front at the same time. This condition would be met in a miscible blend, since miscibility implies that the chains of the component polymers are intimately mixed at the molecular level.^62^ Adding EPPOSS to the 50PA6/50TPE blend, the Δ*H*_c_ values of the samples show values between those of the pure polymers and are higher than the enthalpy of crystallization of TPE alone, suggesting cocrystallization. Moreover, the completion of crystallization in the blends occurs above the final temperature of crystallization of TPE, which indicates that EPPOSS acts as a compatibilizer. The graft and/or block copolymers formed in the existence of EPPOSS suppress the growth phase of the embryo crystals formed.

The melting temperatures (*T*_m_) of pure PA6 and pure TPE were 221.4 and 190.8 °C, respectively. The melting enthalpies (Δ*H*_m_) of pure PA6 and pure TPE were determined as 48.6 and 12.8 J/g, respectively, indicating the presence of a more regular crystal morphology in PA6. The bimodal melting behavior observed in PA6 suggests the existence of different crystal structures. The double melting endotherm is attributed to the polymorphism of PA6, where α and β crystal forms coexist with melting temperatures of 214.0 and 221.4 °C, respectively.^63^ When TPE was added to PA6, only the melting behavior of the PA6 phase was observed, and the melting temperature of PA6 remained unchanged. The bimodal melting behavior of PA6 was eliminated due to the suppression of α crystals by the TPE phase. The absence of melting behavior in the TPE phase of PA6/TPE blends was attributed to the complete suppression of TPE crystals by more stable PA6 crystals upon heating. Only one melting endotherm was observed when MultEpPOSS and EPPOSS were added to the 70PA6/30TPE blend. The presence of POSS has a minimal effect on the melting temperature values of the PA6 phase in the PA6/TPE blends. The existence of MultEpPOSS improves the crystallizability of PA6 by allowing part of the PA6 chains to nucleate and crystallize before TPE. Selective localization analyses verify that MultEpPOSS is localized predominantly in the TPE phase, decreasing the viscoelastic mismatch between the two matrices and supporting the disintegration of TPE droplets in the dispersed phase. The finer and more homogeneous TPE particles act as heterogeneous nucleation sites, resulting in the crystallization of the PA6 phase and enhanced melting enthalpy values. The increasing content of EPPOSS from 0.5 to 1 wt % also increases the melting enthalpy values, indicating a compatibilization efficiency. Pure TPE exhibits a melting temperature range of 167–200 °C, while 50PA6/50TPE blends containing POSS show a melting temperature range of approximately 185–225 °C. This indicates comelting behavior between TPE and PA6 crystals. Moreover, the presence of POSS molecules in 50PA6/50TPE blends, except for the 1 wt % MultEpPOSS blend, results in bimodal melting behavior. The first peak refers to the melting temperature of α crystals (∼205 °C), while the second peak corresponds to the melting temperature of β crystals (∼216 °C). The regeneration of α crystals in the presence of POSS molecules is attributed to the decreased particle size of TPE after compatibilization. The occurrence of a single melting endotherm in the presence of 1 wt % MultEpPOSS is due to the formation of a highly stable phase morphology.

## 4 Conclusions

In this study, the compatibilization effect of POSS nanoparticles with multiple epoxy groups (MultEpPOSS and EPPOSS) on PA6/TPE blends was investigated in various aspects. FTIR analyses revealed the reactions of epoxy groups in POSS with repeating amide groups in the main chain of PA6 by the formation of tertiary amide groups. Also, the disappearance of epoxy groups of POSS nanoparticles in the compatibilized PA6/TPE blends indicates successful interactions between POSS and PA6 and/or TPE. SEM analyses demonstrated a change in the dispersed phase particle effects upon incorporation of POSS into the PA6/TPE blend. Selective localization measurements indicated that POSSs are located in the TPE phase. The addition of POSS to the PA6/TPE blend led to an increase in rheological properties attributed to the formation of PA6-*co*-POSS-*co*-TPE block or graft copolymers at the interface. Tensile tests revealed that the addition of POSS increased the tensile strength and Young’s modulus values while decreasing the elongation at break. Furthermore, the addition of TPE to PA6 led to the elimination of notch sensitivity in PA6, while the addition of POSS to the blends led to a significant increase in Izod impact strength values. Based on the type of POSS, loading level, and PA6/TPE ratio, cocrystallization was observed in the blends, indicating a remarkable improvement in the interfacial interaction between the components. Based on these comprehensive findings, it can be concluded that environmentally friendly POSS nanoparticles can serve as effective compatibilizers for PA6/TPE blends and expand the potential applications of these materials, especially in the automotive industry.

## Supporting Information Available

Average contact angles of the samples used to determine the surface energy values of the components (PDF)
