# Supplementary material for: Effects of Hybrid POSS Nanoparticles on the Properties of Thermoplastic Elastomer-Toughened Polyamide 6
Source: ACS Omega. 2023 Nov 29;8(49):47034–50. doi: 10.1021/acsomega.3c06896 (PMC10720298; doi:10.1021/acsomega.3c06896)
Supplement: Supplementary file 1 — ao3c06896_si_001.pdf [file ao3c06896_si_001.pdf]

Supporting Information

Effects of hybrid POSS nanoparticles on the properties of thermoplastic elastomer toughened polyamide 6

Rumeysa Yıldırım[1], Muhammad Saeed Ullah[2], Hürol Koçoğlu[3], Merve Ün[1], Nazlı Yazıcı Çakır[2], Gülşah Demir[2], Duygu Çetin[1], Gizem Urtekin[2], Güralp Özkoç[4,5], Olcay Mert[1,6], Mehmet Kodal[1,2,4 *]

[1]Polymer Science and Technology Graduate Programme, Kocaeli University, 41001, Kocaeli, Türkiye

[2]Chemical Engineering Department, Kocaeli University, 41001, Kocaeli, Türkiye

[3]Mechanical Engineering Department, Bolu Abant Izzet Baysal University, 14030, Bolu, Türkiye

[4]Nanotechnology Research and Application Center SUNUM, Sabanci University, 34956, İstanbul, Türkiye

[5]Chemistry Department, İstinye University, 34010, İstanbul, Türkiye

[6]Chemistry Department, Kocaeli University, 41001, Kocaeli, Türkiye

[*]Corresponding Author: Mehmet Kodal
mehmet.kodal@kocaeli.edu.tr

Table S1. Average contact angles of probe liquids on the surface of PA6 and TPE

Sample	Contact Angle (θ)		
	Diiodomethane	Ethylene glycol	Deionized water
PA6	32.2±5.2	49.5±3.9	72.8±1.3
TPE	23.1±5.6	51.7±1.7	67.3±1.9

Table S2. Average contact angles of POSSs on probe solids (polymers)

Sample	Contact Angle (θ)		
	PP	PA6	PS
MultEpPOSS	62.8±4.4	59.0±2.5	68.3±5.4
EPPOSS	28.9±1.8	35.4±2.6	42.7±4.7
